# Supplementary material for: Fin whale song evolution in the North Atlantic
Source: eLife. 2024 Jan 9;13:e83750. doi: 10.7554/eLife.83750 (PMC10776088; doi:10.7554/eLife.83750)
Supplement: Supplementary file 1. — (a) Table showing information of each song analysed in this study: date, time of the first (Time first note) and last note analysed (Time last note), duration and number of INIs measured (Num. INIs). (b). Position, recording equipment and approximate (Approx.) depth for each location sampled. (c). Description of fin whale song measurements made with Raven Pro 1.4 software (Charif et al., 2010). (d) The effect of recording equipment on fin whale song parameters When comparing data from multiple sensors an obvious question is whether the results might be dependent on the specific sensors considered. To investigate the influence of the acoustic recorder type, OBS or ARs, on the song parameters, we analysed the same song fragment, consisting of 209 notes, recorded by the hydrophone channel of an OBS and an AR, specifically an Ecological Acoustic Recorder (EAR) (Lammers et al., 2008). The two instruments were deployed at ~6 km from each other in the Azores region in spring of 2019. Measurements of INIs and 20 Hz peak frequencies of songs recorded by each instrument were compared using a non-parametric paired samples Wilcoxon Test. Differences in HF note peak frequencies could not be tested because of limitations in the sampling rate of the OBS. Results showed that median INIs measured from OBS (16.52) and EARs (16.42) were not significantly different (P-value = 0.47) but median peak frequencies of the 20 Hz note (median OBS = 23.4; EARs = 21.1) were (P-value <0.001). Thus, the use of different recorders did not affect INI measurements but influenced measurements of 20 Hz peak frequencies. The effect of the distance to the source in the analysed fin whale song parameters is included in this study, given that these two recorders were positioned at different distances to the singer. Peak frequencies of the 20 Hz note showed a great variability between equipment types (Fig. S3), which hindered the identification of soft trends (i.e., low changing rate). For this reason, only [file elife-83750-supp1.docx]

**Fin whale song evolution in the North Atlantic**

Miriam Romagosa^1^, Sharon Nieukirk^2^, Irma Cascão^1^, Tiago A. Marques^3,4^, Robert Dziak^5^, Jean-Yves Royer^6^, Joanne O’Brien^7^, David K. Mellinger^2,5^, Andreia Pereira^8^, Arantza Ugalde^9^, Elena Papale^10^, Sofia Aniceto^11^, Giuseppa Buscaino^10^, Marianne Rasmussen^12^, Luis Matias^8^, Rui Prieto^1^ & Mónica A. Silva^1^

^1^ Institute of Marine Sciences – OKEANOS & Institute of Marine Research – IMAR, University of the Azores, Horta, Portugal; ^2^Cooperative Institute for Marine Ecosystem and Resources Studies, Oregon State University, Oregon, USA; ^3^Centre for Research into Ecological and Environmental Modelling, University of St Andrews, St Andrews, UK; ^4^Centro de Estatística e Aplicações, Departamento de Biologia, Faculdade de Ciências, Universidade de Lisboa, Lisboa, Portugal; ^5^NOAA Pacific Marine Environmental Laboratory, Hatfield Marine Science Center, Oregon, USA; ^6^Univ Brest, CNRS, Laboratoire Geosciences Ocean, Plouzane, France.^7^Marine and Freshwater Research Centre, Galway-Mayo Institute of Technology, Galway, Ireland; ^8^Instituto Dom Luiz (IDL), Universidade de Lisboa, Lisboa, Portugal; ^9^Institute of Marine Sciences, ICM‐CSIC, Barcelona, Spain; ^10^Institute for the Study of Anthropic Impacts and Sustainability in the Marine Environment of the National Research Council of Italy (CNR-IAS), Torretta Granitola, Italy; ^11^Akvaplan Niva, Tromsø, Norway; ^12^University of Iceland’s research center in Húsavík, Iceland, Iceland.

**SUPPLEMENTARY MATERIAL**

Supplementary file 1a. Table showing information of each song analysed in this study: date, time of the first (Time first note) and last note analysed (Time last note), duration and number of INIs measured (Num. INIs).

| Region | Location | Date | Time first note | Duration (h) | Time last note | Num. INIs |
| --- | --- | --- | --- | --- | --- | --- |
| Barents Sea | Vesterålen | 02/01/2018 | 00:03:52 | 0.3 | 00:18:55 | 69 |
|  |  | 09/01/2018 | 14:58:02 | 0.5 | 10:18:47 | 113 |
|  |  | 25/01/2018 | 23:27:32 | 0.6 | 00:02:42 | 173 |
|  |  | 26/01/2018 | 00:45:16 | 0.2 | 00:16:11 | 46 |
|  |  | 18/02/2018 | 06:57:39 | 0.5 | 06:59:05 | 125 |
|  |  | 21/02/2018 | 14:52:32 | 1.4 | 09:58:08 | 153 |
|  |  | 23/02/2018 | 12:02:42 | 0.9 | 12:52:13 | 129 |
|  |  | 26/02/2018 | 13:26:11 | 0.7 | 12:27:43 | 119 |
|  | Svalbard | 09/10/2014 | 22:04:28 | 0.4 | 22:26:33 | 96 |
|  |  | 18/10/2014 | 02:16:24 | 0.2 | 02:12:18 | 50 |
|  |  | 20/10/2014 | 00:10:59 | 0.2 | 00:13:27 | 45 |
|  |  | 08/10/2015 | 14:02:33 | 0.1 | 14:03:25 | 13 |
|  |  | 13/11/2015 | 13:21:41 | 0.1 | 13:08:17 | 19 |
|  |  | 12/01/2016 | 02:00:30 | 0.5 | 02:28:56 | 37 |
| Canary Islands | Canary Islands | 02/11/2014 | 00:00:08 | 1.0 | 00:59:45 | 125 |
|  |  | 05/11/2014 | 02:25:30 | 0.9 | 00:53:53 | 121 |
|  |  | 08/11/2014 | 03:08:23 | 17.6 | 17:36:11 | 114 |
|  |  | 17/11/2014 | 16:50:00 | 6.0 | 05:58:47 | 52 |
|  |  | 20/11/2014 | 06:02:29 | 0.5 | 00:29:43 | 73 |
|  |  | 23/11/2014 | 00:41:14 | 0.9 | 00:54:20 | 53 |
|  |  | 26/11/2014 | 19:32:49 | 0.2 | 00:13:28 | 55 |
|  |  | 29/11/2014 | 04:11:35 | 0.4 | 00:26:18 | 72 |
|  |  | 14/12/2014 | 00:00:10 | 1.6 | 01:37:24 | 99 |
|  |  | 17/12/2014 | 07:07:34 | 0.7 | 00:44:56 | 130 |
|  |  | 20/12/2014 | 03:55:41 | 0.6 | 00:37:31 | 77 |
|  |  | 29/12/2014 | 04:46:27 | 0.6 | 00:35:38 | 124 |
|  |  | 31/12/2014 | 16:12:18 | 0.6 | 00:36:13 | 96 |
|  |  | 03/01/2015 | 22:05:10 | 0.3 | 00:18:20 | 58 |
|  |  | 06/01/2015 | 00:09:29 | 4.8 | 04:46:43 | 54 |
|  |  | 12/01/2015 | 07:40:00 | 0.5 | 00:31:42 | 105 |
|  |  | 18/01/2015 | 22:28:58 | 1.0 | 00:57:40 | 92 |
|  |  | 21/01/2015 | 15:09:51 | 0.8 | 00:47:20 | 164 |
|  |  | 26/01/2015 | 13:39:20 | 0.7 | 00:41:56 | 100 |
|  |  | 28/01/2015 | 13:49:59 | 1.1 | 01:04:59 | 91 |
|  |  | 30/01/2015 | 18:32:20 | 0.8 | 00:50:28 | 136 |
| Celtic Sea | North Porcupine | 01/10/2015 | 22:41:09 | 0.5 | 00:02:00 | 52 |
|  |  | 06/10/2015 | 14:37:22 | 0.9 | 00:02:00 | 152 |
|  |  | 20/10/2015 | 13:59:21 | 0.8 | 00:02:00 | 82 |
|  | South Porcupine | 20/03/2016 | 22:02:10 | 0.5 | 00:02:00 | 64 |
|  |  | 04/10/2016 | 06:27:41 | 0.8 | 00:02:00 | 97 |
|  |  | 08/10/2016 | 00:17:51 | 0.2 | 00:02:00 | 29 |
|  |  | 12/10/2016 | 09:46:47 | 0.5 | 00:02:00 | 71 |
|  |  | 18/10/2016 | 19:19:33 | 1.0 | 00:02:00 | 132 |
|  |  | 21/10/2016 | 07:15:47 | 0.7 | 00:02:00 | 107 |
|  |  | 29/10/2016 | 02:31:28 | 0.6 | 00:02:00 | 83 |
|  |  | 02/11/2016 | 16:21:25 | 0.8 | 00:02:00 | 91 |
| SE Greenland | SE Greenland | 08/10/2007 | 13:02:19 | 0.1 | 12:28:54 | 23 |
|  |  | 21/10/2007 | 03:43:00 | 0.3 | 03:53:51 | 49 |
|  |  | 24/10/2007 | 21:47:25 | 0.5 | 22:17:16 | 89 |
|  |  | 31/10/2007 | 09:32:28 | 0.9 | 10:23:06 | 123 |
|  |  | 03/11/2007 | 03:07:52 | 1.0 | 04:04:47 | 82 |
|  |  | 08/11/2007 | 14:17:18 | 0.9 | 15:09:16 | 146 |
|  |  | 17/11/2007 | 03:11:07 | 0.2 | 03:20:18 | 18 |
|  |  | 01/12/2007 | 00:39:06 | 0.7 | 20:18:05 | 149 |
|  |  | 04/12/2007 | 00:41:58 | 1.5 | 22:55:48 | 301 |
|  |  | 12/12/2007 | 08:08:18 | 1.5 | 02:57:50 | 293 |
|  |  | 28/12/2007 | 07:40:09 | 1.2 | 03:58:31 | 219 |
|  |  | 05/01/2008 | 23:53:01 | 1.4 | 01:15:20 | 177 |
|  |  | 12/01/2008 | 11:36:21 | 0.9 | 12:31:19 | 121 |
|  |  | 17/01/2008 | 16:35:58 | 1.5 | 16:05:19 | 265 |
|  |  | 26/01/2008 | 09:33:06 | 1.3 | 04:46:40 | 219 |
|  |  | 02/02/2008 | 17:25:33 | 1.2 | 17:00:00 | 225 |
|  |  | 16/02/2008 | 15:20:14 | 0.5 | 08:09:51 | 98 |
|  |  | 22/02/2008 | 05:08:53 | 0.4 | 03:25:56 | 49 |
|  |  | 26/02/2008 | 04:09:20 | 0.9 | 22:37:07 | 83 |
|  |  | 03/03/2008 | 22:56:45 | 0.6 | 17:40:08 | 77 |
|  |  | 07/03/2008 | 09:48:31 | 0.4 | 03:25:48 | 19 |
|  |  | 12/03/2008 | 06:25:47 | 0.2 | 22:33:45 | 16 |
| SW Portugal | SW Portugal | 01/12/2007 | 02:07:43 | 0.3 | 00:16:25 | 61 |
|  |  | 11/12/2007 | 20:57:29 | 2.4 | 02:23:00 | 25 |
|  |  | 21/12/2007 | 02:36:48 | 0.2 | 00:10:35 | 21 |
|  |  | 25/12/2007 | 05:37:29 | 1.8 | 01:50:45 | 71 |
|  |  | 07/01/2008 | 03:53:35 | 14.4 | 14:24:02 | 67 |
|  |  | 11/01/2008 | 02:03:33 | 3.4 | 03:23:50 | 25 |
|  |  | 16/01/2008 | 22:20:55 | 1.5 | 01:28:23 | 44 |
|  |  | 24/01/2008 | 21:50:30 | 1.0 | 00:59:36 | 25 |
|  |  | 27/01/2008 | 08:42:59 | 1.8 | 01:45:56 | 117 |
|  |  | 31/01/2008 | 08:48:41 | 13.5 | 13:30:26 | 23 |
|  |  | 03/02/2008 | 23:14:36 | 0.6 | 00:38:14 | 23 |
|  |  | 19/02/2008 | 10:02:13 | 1.9 | 01:56:21 | 161 |
|  |  | 22/02/2008 | 17:37:06 | 2.3 | 02:16:38 | 34 |
|  |  | 29/02/2008 | 03:21:06 | 1.8 | 01:47:07 | 121 |
|  |  | 08/10/2015 | 02:31:42 | 0.4 | 00:02:00 | 38 |
|  |  | 12/10/2015 | 19:45:13 | 0.1 | 00:02:00 | 14 |
|  |  | 24/10/2015 | 03:30:17 | 0.4 | 00:02:00 | 41 |
|  |  | 31/10/2015 | 03:30:14 | 0.2 | 00:02:00 | 16 |
|  |  | 06/11/2015 | 05:42:17 | 1.2 | 00:02:00 | 124 |
|  |  | 06/12/2015 | 00:00:08 | 0.6 | 00:02:00 | 86 |
|  |  | 10/12/2015 | 16:45:06 | 0.8 | 00:02:00 | 116 |
|  |  | 12/12/2015 | 13:15:06 | 0.3 | 00:02:00 | 38 |
|  |  | 15/12/2015 | 00:15:18 | 0.9 | 00:02:00 | 58 |
|  |  | 18/12/2015 | 04:15:17 | 0.3 | 00:02:00 | 48 |
|  |  | 20/12/2015 | 21:30:04 | 0.1 | 00:02:00 | 21 |
|  |  | 28/12/2015 | 23:17:05 | 0.1 | 00:02:00 | 14 |
|  |  | 04/01/2016 | 11:00:07 | 0.3 | 00:02:00 | 43 |
|  |  | 06/01/2016 | 01:45:12 | 0.3 | 00:02:00 | 19 |
|  |  | 08/01/2016 | 16:45:13 | 0.1 | 00:02:00 | 24 |
|  |  | 10/01/2016 | 05:45:06 | 0.1 | 00:02:00 | 22 |
|  |  | 12/01/2016 | 19:30:37 | 0.3 | 00:02:00 | 63 |
|  |  | 15/01/2016 | 17:15:16 | 0.3 | 00:02:00 | 51 |
|  |  | 17/01/2016 | 10:01:07 | 0.3 | 00:02:00 | 28 |
|  |  | 19/01/2016 | 16:30:11 | 0.1 | 00:02:00 | 29 |
|  |  | 21/01/2016 | 07:30:09 | 0.2 | 00:02:00 | 31 |
|  |  | 24/01/2016 | 21:45:06 | 0.2 | 00:02:00 | 39 |
|  |  | 01/02/2016 | 17:00:10 | 0.2 | 00:02:00 | 29 |
|  |  | 03/02/2016 | 19:30:09 | 0.3 | 00:02:00 | 31 |
|  |  | 17/02/2016 | 04:16:21 | 0.3 | 00:02:00 | 50 |
|  |  | 25/02/2016 | 10:45:15 | 0.2 | 00:02:00 | 18 |
|  |  | 27/02/2016 | 21:45:08 | 0.1 | 00:02:00 | 20 |
|  |  | 06/03/2016 | 11:30:11 | 0.6 | 00:02:00 | 84 |
| SE Iceland | SE Iceland | 15/02/2007 | 13:11:58 | 0.4 | 20:02:44 | 48 |
|  |  | 17/02/2007 | 20:19:07 | 1.7 | 00:09:04 | 136 |
|  |  | 11/03/2007 | 18:01:36 | 1.4 | 20:36:14 | 107 |
| ONA | Azores | 12/10/2008 | 04:40:54 | 0.2 | 00:02:00 | 11 |
|  |  | 17/10/2008 | 17:32:06 | 0.2 | 00:02:00 | 26 |
|  |  | 23/10/2008 | 14:59:19 | 0.8 | 00:02:00 | 18 |
|  |  | 03/11/2008 | 01:45:54 | 0.3 | 00:02:00 | 22 |
|  |  | 25/11/2008 | 07:12:08 | 0.3 | 00:02:00 | 56 |
|  |  | 29/11/2008 | 18:22:50 | 0.2 | 00:02:00 | 25 |
|  |  | 06/12/2008 | 13:21:15 | 0.5 | 00:02:00 | 15 |
|  |  | 09/12/2008 | 08:54:31 | 1.2 | 00:02:00 | 22 |
|  |  | 16/12/2008 | 16:05:47 | 0.3 | 00:02:00 | 32 |
|  |  | 04/10/2009 | 05:52:38 | 0.8 | 00:02:00 | 65 |
|  |  | 20/10/2009 | 01:54:15 | 0.2 | 00:02:00 | 21 |
|  |  | 29/10/2009 | 22:42:16 | 0.2 | 00:02:00 | 37 |
|  |  | 27/10/2010 | 04:19:45 | 0.2 | 00:02:00 | 22 |
|  |  | 04/11/2010 | 22:16:48 | 0.2 | 00:02:00 | 23 |
|  |  | 06/11/2010 | 05:52:48 | 0.3 | 00:02:00 | 29 |
|  |  | 21/11/2010 | 19:14:15 | 0.5 | 00:02:00 | 20 |
|  |  | 24/11/2010 | 17:04:55 | 0.1 | 00:02:00 | 16 |
|  |  | 28/11/2010 | 21:00:30 | 0.4 | 00:02:00 | 36 |
|  |  | 03/12/2010 | 12:15:09 | 0.4 | 00:02:00 | 15 |
|  |  | 14/12/2010 | 22:24:11 | 0.1 | 00:02:00 | 12 |
|  |  | 17/12/2010 | 04:30:38 | 0.3 | 00:02:00 | 19 |
|  |  | 23/12/2010 | 16:34:51 | 0.2 | 00:02:00 | 19 |
|  |  | 29/12/2010 | 13:49:33 | 1.3 | 00:02:00 | 29 |
|  |  | 30/12/2010 | 11:06:53 | 0.2 | 00:02:00 | 17 |
|  |  | 03/01/2011 | 08:19:34 | 0.2 | 00:02:00 | 20 |
|  |  | 07/01/2011 | 09:21:17 | 0.6 | 00:02:00 | 22 |
|  |  | 09/01/2011 | 19:07:59 | 0.4 | 00:02:00 | 16 |
|  |  | 11/01/2011 | 09:30:26 | 0.4 | 00:02:00 | 37 |
|  |  | 30/01/2011 | 06:08:00 | 0.1 | 00:02:00 | 25 |
|  |  | 11/02/2011 | 09:58:04 | 0.2 | 00:02:00 | 18 |
|  |  | 27/10/2011 | 20:42:15 | 0.6 | 21:16:36 | 32 |
|  |  | 08/11/2011 | 18:15:44 | 0.5 | 18:25:34 | 44 |
|  |  | 20/11/2011 | 03:36:13 | 0.1 | 03:43:42 | 12 |
|  |  | 01/12/2011 | 20:30:27 | 0.7 | 20:53:41 | 119 |
|  |  | 06/12/2011 | 15:14:22 | 1.0 | 16:09:43 | 54 |
|  |  | 12/12/2011 | 16:23:40 | 0.7 | 16:47:17 | 89 |
|  |  | 19/12/2011 | 07:16:35 | 0.5 | 07:19:32 | 28 |
|  |  | 03/01/2012 | 15:13:48 | 0.2 | 14:57:54 | 21 |
|  |  | 06/01/2012 | 21:10:23 | 0.6 | 21:35:39 | 78 |
|  |  | 12/01/2012 | 19:37:45 | 0.5 | 20:06:53 | 59 |
|  |  | 17/01/2012 | 17:01:48 | 0.7 | 17:33:55 | 52 |
|  |  | 20/01/2012 | 20:48:17 | 1.0 | 21:47:48 | 160 |
|  |  | 04/02/2012 | 19:52:09 | 0.4 | 17:43:59 | 53 |
|  |  | 11/02/2012 | 02:54:03 | 0.7 | 22:29:20 | 30 |
|  |  | 14/02/2012 | 11:34:35 | 0.3 | 05:17:49 | 20 |
|  |  | 25/02/2012 | 10:16:12 | 0.8 | 04:02:56 | 82 |
|  |  | 02/03/2012 | 06:27:43 | 0.9 | 07:19:01 | 65 |
|  |  | 04/03/2012 | 08:28:53 | 0.1 | 06:47:53 | 19 |
|  |  | 07/10/2012 | 10:08:27 | 0.1 | 09:08:41 | 12 |
|  |  | 24/02/2017 | 08:00:23 | 6.0 | 13:59:33 | 82 |
|  |  | 27/02/2017 | 08:03:40 | 0.2 | 08:14:50 | 35 |
|  |  | 02/03/2017 | 08:47:51 | 3.6 | 11:33:09 | 181 |
|  |  | 06/03/2017 | 11:18:20 | 0.9 | 08:52:47 | 140 |
|  |  | 09/03/2017 | 11:10:15 | 2.6 | 10:34:17 | 89 |
|  |  | 12/03/2017 | 09:51:46 | 3.7 | 11:43:14 | 51 |
|  |  | 16/03/2017 | 12:56:36 | 0.3 | 08:16:58 | 18 |
|  |  | 18/03/2017 | 08:48:34 | 5.2 | 13:11:24 | 106 |
|  |  | 26/03/2017 | 10:54:16 | 0.7 | 08:44:32 | 55 |
|  |  | 18/12/2017 | 11:12:58 | 3.2 | 12:11:11 | 345 |
|  |  | 08/01/2018 | 12:12:58 | 0.4 | 09:22:18 | 64 |
|  |  | 11/01/2018 | 09:02:18 | 1.2 | 10:10:28 | 113 |
|  |  | 13/01/2018 | 09:32:04 | 0.1 | 09:08:06 | 22 |
|  |  | 20/03/2019 | 11:56:40 | 0.3 | 08:16:59 | 48 |
|  |  | 22/12/2019 | 17:29:55 | 1.6 | 15:33:16 | 43 |
|  |  | 01/01/2020 | 15:07:35 | 0.4 | 14:23:37 | 52 |
|  |  | 24/01/2020 | 14:00:12 | 1.7 | 15:42:37 | 81 |
|  |  | 31/01/2020 | 18:18:24 | 1.5 | 15:31:57 | 114 |
|  |  | 03/02/2020 | 17:18:24 | 2.7 | 16:40:46 | 64 |
|  |  | 05/02/2020 | 14:00:28 | 4.3 | 18:18:50 | 297 |
|  |  | 13/02/2020 | 14:26:22 | 0.7 | 14:39:11 | 31 |
|  |  | 22/02/2020 | 17:33:17 | 2.1 | 16:07:04 | 25 |
|  |  | 27/02/2020 | 10:32:57 | 1.0 | 09:00:29 | 158 |
|  |  | 11/03/2020 | 12:57:13 | 0.4 | 08:22:54 | 47 |
|  |  | 17/03/2020 | 09:42:11 | 0.9 | 08:52:57 | 59 |
|  |  | 21/03/2020 | 08:54:30 | 0.4 | 08:25:12 | 62 |
|  |  | 06/01/2006 | 00:00:50 | 0.9 | 00:54:51 | 161 |
|  |  | 10/01/2007 | 00:19:26 | 0.2 | 00:13:01 | 41 |
|  |  | 31/01/2007 | 02:01:23 | 0.6 | 00:34:19 | 94 |
|  |  | 15/01/2008 | 03:10:21 | 1.4 | 01:22:22 | 177 |
|  |  | 20/01/2008 | 10:02:44 | 0.5 | 00:32:32 | 100 |
|  | CE | 01/10/2002 | 14:22:49 | 0.8 | 15:12:20 | 178 |
|  |  | 15/10/2002 | 02:44:58 | 0.7 | 03:28:34 | 119 |
|  |  | 30/10/2002 | 12:47:58 | 1.1 | 12:52:14 | 148 |
|  |  | 02/11/2002 | 01:16:26 | 1.4 | 03:24:04 | 263 |
|  |  | 11/11/2002 | 09:06:21 | 0.7 | 10:13:57 | 135 |
|  |  | 24/11/2002 | 06:12:16 | 0.5 | 06:43:28 | 122 |
|  |  | 30/11/2002 | 18:43:54 | 1.0 | 20:45:48 | 81 |
|  |  | 05/12/2002 | 04:40:30 | 0.7 | 05:21:19 | 114 |
|  |  | 12/12/2002 | 20:42:25 | 0.8 | 21:56:20 | 188 |
|  |  | 30/12/2002 | 23:05:43 | 0.9 | 00:16:09 | 202 |
|  |  | 10/01/2003 | 16:55:16 | 0.7 | 17:31:31 | 82 |
|  |  | 15/01/2003 | 18:53:08 | 1.0 | 20:04:10 | 136 |
|  |  | 24/01/2003 | 16:12:18 | 1.2 | 18:05:56 | 175 |
|  |  | 28/01/2003 | 14:18:49 | 0.7 | 14:48:10 | 69 |
|  |  | 02/02/2003 | 21:23:17 | 1.0 | 23:20:46 | 130 |
|  |  | 13/02/2003 | 22:29:48 | 0.6 | 23:03:10 | 132 |
|  |  | 05/03/2003 | 08:09:44 | 0.5 | 08:07:16 | 72 |
|  |  | 10/03/2003 | 23:12:43 | 0.6 | 23:26:32 | 76 |
|  |  | 17/03/2003 | 02:18:29 | 0.7 | 03:05:26 | 143 |
|  |  | 30/03/2003 | 18:45:42 | 1.6 | 21:22:33 | 252 |
|  | CW | 09/10/2002 | 16:55:02 | 1.1 | 18:15:07 | 59 |
|  |  | 17/10/2002 | 20:34:41 | 0.9 | 22:10:08 | 135 |
|  |  | 22/10/2002 | 02:15:59 | 0.5 | 02:40:58 | 65 |
|  |  | 30/10/2002 | 07:53:34 | 1.0 | 09:20:25 | 77 |
|  |  | 02/11/2002 | 12:10:47 | 1.1 | 13:18:22 | 191 |
|  |  | 16/11/2002 | 08:33:31 | 1.5 | 10:44:56 | 231 |
|  |  | 25/11/2002 | 09:03:38 | 1.2 | 11:15:50 | 160 |
|  |  | 30/11/2002 | 13:38:45 | 1.1 | 15:23:23 | 164 |
|  |  | 05/12/2002 | 03:28:16 | 0.8 | 04:57:34 | 145 |
|  |  | 11/12/2002 | 18:35:40 | 0.7 | 18:59:59 | 85 |
|  |  | 14/12/2002 | 01:40:58 | 0.7 | 01:21:37 | 94 |
|  |  | 23/12/2002 | 10:54:37 | 0.5 | 11:33:23 | 72 |
|  |  | 05/01/2003 | 07:15:34 | 1.3 | 09:02:57 | 147 |
|  |  | 07/01/2003 | 19:25:29 | 0.5 | 19:35:22 | 83 |
|  |  | 16/01/2003 | 12:28:14 | 0.7 | 13:33:28 | 132 |
|  |  | 28/01/2003 | 20:13:41 | 0.9 | 21:11:44 | 150 |
|  |  | 05/02/2003 | 01:56:59 | 0.9 | 03:01:54 | 217 |
|  |  | 08/02/2003 | 20:59:38 | 1.1 | 22:27:42 | 134 |
|  |  | 18/02/2003 | 18:02:26 | 0.7 | 19:10:22 | 64 |
|  |  | 22/02/2003 | 17:52:41 | 0.6 | 18:16:51 | 39 |
|  |  | 04/03/2003 | 00:09:24 | 1.1 | 02:15:40 | 100 |
|  |  | 11/03/2003 | 03:49:06 | 1.3 | 05:45:56 | 82 |
|  |  | 16/03/2003 | 05:11:23 | 1.2 | 07:16:00 | 174 |
|  |  | 30/03/2003 | 09:23:29 | 1.3 | 21:04:13 | 130 |
|  | NE | 01/10/2002 | 11:22:31 | 1.0 | 12:40:50 | 185 |
|  |  | 25/10/2002 | 06:32:04 | 0.6 | 07:28:55 | 84 |
|  |  | 29/10/2002 | 01:06:16 | 1.2 | 03:18:43 | 232 |
|  |  | 31/10/2002 | 23:14:48 | 0.6 | 22:39:28 | 68 |
|  |  | 10/11/2002 | 17:16:34 | 1.8 | 19:57:18 | 63 |
|  |  | 13/11/2002 | 03:35:21 | 1.1 | 05:34:45 | 197 |
|  |  | 20/11/2002 | 20:09:41 | 1.1 | 22:00:09 | 160 |
|  |  | 28/11/2002 | 08:56:52 | 0.3 | 09:01:29 | 44 |
|  |  | 01/12/2002 | 23:41:54 | 0.3 | 23:19:36 | 66 |
|  |  | 02/12/2002 | 01:00:50 | 0.6 | 00:35:27 | 123 |
|  |  | 05/12/2002 | 05:41:00 | 1.1 | 07:00:16 | 177 |
|  |  | 19/12/2002 | 09:22:09 | 0.9 | 10:30:23 | 195 |
|  |  | 03/01/2003 | 15:05:09 | 0.6 | 15:35:22 | 127 |
|  |  | 07/01/2003 | 19:04:42 | 0.7 | 19:53:59 | 74 |
|  |  | 14/01/2003 | 06:10:24 | 0.8 | 07:10:54 | 104 |
|  |  | 23/01/2003 | 21:04:45 | 1.4 | 22:55:28 | 117 |
|  |  | 04/02/2003 | 06:36:11 | 1.2 | 08:49:23 | 125 |
|  |  | 07/02/2003 | 17:43:43 | 0.4 | 17:37:08 | 74 |
|  |  | 12/02/2003 | 05:50:26 | 0.5 | 06:33:57 | 80 |
|  |  | 23/02/2003 | 17:18:53 | 1.1 | 18:53:22 | 126 |
|  |  | 04/03/2003 | 22:25:53 | 0.5 | 21:44:32 | 37 |
|  |  | 18/03/2003 | 09:29:14 | 0.6 | 10:14:42 | 63 |
|  |  | 26/03/2003 | 12:23:24 | 1.0 | 13:49:26 | 129 |
|  | NW | 06/10/2002 | 10:56:28 | 0.8 | 11:41:28 | 78 |
|  |  | 12/10/2002 | 09:35:30 | 0.3 | 10:02:41 | 34 |
|  |  | 17/10/2002 | 13:54:31 | 0.6 | 14:29:44 | 51 |
|  |  | 28/10/2002 | 13:57:11 | 0.9 | 14:45:35 | 175 |
|  |  | 10/11/2002 | 03:10:00 | 1.2 | 04:59:52 | 256 |
|  |  | 19/11/2002 | 09:30:01 | 1.1 | 11:13:37 | 138 |
|  |  | 23/11/2002 | 15:21:34 | 1.6 | 17:38:06 | 112 |
|  |  | 04/12/2002 | 05:05:30 | 0.8 | 06:30:32 | 137 |
|  |  | 08/12/2002 | 18:32:06 | 1.2 | 20:23:15 | 210 |
|  |  | 15/12/2002 | 21:04:34 | 0.7 | 22:02:02 | 115 |
|  |  | 31/12/2002 | 09:09:51 | 0.8 | 09:56:04 | 160 |
|  |  | 02/01/2003 | 07:05:27 | 0.6 | 07:21:37 | 86 |
|  |  | 04/01/2003 | 06:50:32 | 0.5 | 06:49:28 | 45 |
|  |  | 18/01/2003 | 11:51:42 | 1.0 | 12:51:28 | 53 |
|  |  | 25/01/2003 | 13:22:49 | 0.7 | 14:41:17 | 26 |
|  |  | 08/02/2003 | 14:36:13 | 0.6 | 15:24:47 | 43 |
|  |  | 12/02/2003 | 20:09:53 | 0.6 | 21:21:18 | 107 |
|  |  | 21/02/2003 | 02:22:00 | 1.1 | 03:52:43 | 217 |
|  |  | 24/02/2003 | 13:13:36 | 0.7 | 14:04:32 | 105 |
|  |  | 02/03/2003 | 03:21:22 | 0.6 | 04:07:36 | 44 |
|  |  | 10/03/2003 | 16:01:53 | 1.3 | 18:30:52 | 192 |
|  |  | 14/03/2003 | 12:47:11 | 1.3 | 14:41:01 | 170 |
|  |  | 29/03/2003 | 21:23:36 | 1.5 | 23:42:46 | 200 |
|  | SE | 11/02/1999 | 18:07:56 | 3.1 | 22:27:52 | 160 |
|  |  | 19/02/1999 | 10:34:48 | 0.8 | 11:11:25 | 95 |
|  |  | 21/02/1999 | 21:37:37 | 0.7 | 22:17:35 | 90 |
|  |  | 26/02/1999 | 02:46:33 | 0.9 | 03:30:33 | 67 |
|  |  | 02/03/1999 | 16:15:48 | 0.5 | 16:43:18 | 56 |
|  |  | 08/03/1999 | 08:16:17 | 0.6 | 08:28:36 | 58 |
|  |  | 11/03/1999 | 04:39:43 | 0.9 | 06:16:11 | 81 |
|  |  | 13/03/1999 | 06:35:12 | 1.0 | 08:02:06 | 72 |
|  |  | 21/10/1999 | 10:57:16 | 2.0 | 14:12:43 | 304 |
|  |  | 23/10/1999 | 19:29:29 | 1.5 | 22:23:55 | 196 |
|  |  | 27/10/1999 | 17:52:25 | 1.9 | 21:34:26 | 179 |
|  |  | 29/10/1999 | 13:39:18 | 1.1 | 14:53:01 | 141 |
|  |  | 05/11/1999 | 12:21:22 | 1.6 | 14:54:36 | 160 |
|  |  | 08/11/1999 | 04:54:17 | 1.0 | 06:32:14 | 84 |
|  |  | 26/11/1999 | 07:32:38 | 0.9 | 07:34:18 | 116 |
|  |  | 29/11/1999 | 17:31:03 | 0.9 | 18:34:57 | 106 |
|  |  | 09/12/1999 | 01:09:44 | 1.4 | 03:35:07 | 186 |
|  |  | 12/12/1999 | 21:41:52 | 1.8 | 00:43:46 | 267 |
|  |  | 14/12/1999 | 01:17:38 | 0.8 | 02:06:54 | 84 |
|  |  | 30/12/1999 | 01:21:14 | 2.0 | 04:42:51 | 220 |
|  |  | 14/01/2000 | 03:55:25 | 1.8 | 07:01:45 | 257 |
|  |  | 17/01/2000 | 10:35:13 | 0.8 | 11:45:44 | 105 |
|  |  | 22/01/2000 | 02:57:14 | 0.7 | 03:31:19 | 93 |
|  |  | 28/01/2000 | 11:43:51 | 1.8 | 14:21:37 | 263 |
|  |  | 06/02/2000 | 03:58:02 | 0.7 | 04:39:09 | 94 |
|  |  | 11/02/2000 | 03:56:33 | 1.1 | 05:11:42 | 135 |
|  |  | 20/02/2000 | 08:58:55 | 1.2 | 10:30:05 | 147 |
|  |  | 23/02/2000 | 16:20:59 | 0.8 | 17:08:50 | 186 |
|  |  | 01/03/2000 | 18:08:09 | 1.0 | 19:54:26 | 123 |
|  |  | 05/03/2000 | 11:25:23 | 1.0 | 12:24:17 | 109 |
|  |  | 08/03/2000 | 16:55:21 | 1.5 | 18:53:16 | 215 |
|  |  | 17/03/2000 | 05:50:54 | 0.9 | 07:05:52 | 44 |
|  |  | 22/10/2000 | 20:33:20 | 1.5 | 22:54:09 | 216 |
|  |  | 30/10/2000 | 20:00:14 | 0.9 | 20:50:28 | 215 |
|  |  | 06/11/2000 | 14:58:29 | 1.2 | 16:28:42 | 143 |
|  |  | 20/11/2000 | 06:35:45 | 0.9 | 07:43:29 | 131 |
|  |  | 23/11/2000 | 02:24:10 | 0.6 | 02:51:05 | 82 |
|  |  | 28/11/2000 | 01:07:35 | 0.3 | 00:39:21 | 37 |
|  |  | 01/12/2000 | 01:52:10 | 1.5 | 04:37:30 | 125 |
|  |  | 04/12/2000 | 11:46:04 | 2.3 | 15:23:53 | 149 |
|  |  | 21/12/2000 | 10:51:49 | 0.6 | 11:17:36 | 106 |
|  |  | 30/12/2000 | 16:53:29 | 2.3 | 21:25:40 | 219 |
|  |  | 05/01/2001 | 10:05:04 | 1.5 | 12:07:07 | 224 |
|  |  | 07/01/2001 | 22:17:29 | 0.6 | 23:23:43 | 66 |
|  |  | 23/01/2001 | 14:28:30 | 0.9 | 15:42:06 | 87 |
|  |  | 25/01/2001 | 01:00:00 | 0.6 | 01:55:05 | 67 |
|  |  | 07/02/2001 | 01:29:36 | 1.9 | 04:15:16 | 259 |
|  |  | 14/02/2001 | 13:40:28 | 2.3 | 17:47:44 | 82 |
|  |  | 16/02/2001 | 09:04:25 | 1.2 | 10:46:44 | 109 |
|  |  | 20/02/2001 | 19:58:27 | 0.7 | 20:55:39 | 93 |
|  |  | 03/03/2001 | 15:40:22 | 1.3 | 17:33:32 | 149 |
|  |  | 18/03/2001 | 02:20:39 | 0.6 | 03:30:20 | 87 |
|  |  | 26/03/2001 | 14:49:10 | 1.2 | 16:54:23 | 64 |
|  |  | 29/03/2001 | 18:07:12 | 1.9 | 21:22:00 | 119 |
|  |  | 23/10/2002 | 07:01:51 | 1.2 | 07:29:03 | 160 |
|  |  | 12/11/2002 | 11:05:57 | 0.6 | 12:13:35 | 97 |
|  |  | 17/11/2002 | 18:51:34 | 1.4 | 21:14:06 | 205 |
|  |  | 19/11/2002 | 22:59:35 | 1.0 | 00:25:55 | 132 |
|  |  | 08/12/2002 | 06:09:09 | 1.2 | 08:20:28 | 259 |
|  |  | 14/12/2002 | 09:22:20 | 0.7 | 10:40:21 | 113 |
|  |  | 26/12/2002 | 20:28:52 | 0.7 | 21:34:33 | 107 |
|  |  | 28/12/2002 | 00:12:38 | 2.2 | 04:04:43 | 269 |
|  |  | 17/01/2003 | 09:03:41 | 1.0 | 10:34:41 | 198 |
|  |  | 19/01/2003 | 00:10:52 | 0.8 | 00:59:00 | 102 |
|  |  | 23/01/2003 | 18:13:06 | 0.8 | 19:28:02 | 136 |
|  |  | 26/01/2003 | 19:36:12 | 1.0 | 20:35:23 | 123 |
|  |  | 06/02/2003 | 06:32:37 | 1.1 | 08:23:36 | 201 |
|  |  | 09/02/2003 | 10:02:13 | 1.3 | 12:08:57 | 182 |
|  |  | 15/02/2003 | 00:33:25 | 1.4 | 03:02:53 | 231 |
|  |  | 22/02/2003 | 22:06:43 | 0.9 | 23:00:09 | 82 |
|  |  | 02/03/2003 | 06:29:24 | 1.3 | 08:09:48 | 77 |
|  |  | 07/03/2003 | 03:23:16 | 1.1 | 05:26:12 | 104 |
|  |  | 19/03/2003 | 05:07:42 | 0.5 | 06:11:47 | 63 |
|  |  | 28/03/2003 | 08:47:19 | 0.5 | 09:32:34 | 70 |
|  |  | 09/10/2004 | 01:43:32 | 1.0 | 03:35:29 | 134 |
|  |  | 03/11/2004 | 11:08:09 | 1.9 | 14:01:06 | 222 |
|  |  | 12/11/2004 | 05:02:26 | 1.5 | 07:18:22 | 222 |
|  |  | 20/11/2004 | 07:29:12 | 1.4 | 09:42:35 | 299 |
|  |  | 29/11/2004 | 19:57:07 | 1.1 | 21:25:15 | 141 |
|  |  | 02/12/2004 | 22:25:35 | 0.8 | 00:01:21 | 123 |
|  |  | 10/12/2004 | 01:50:56 | 0.5 | 02:25:25 | 85 |
|  |  | 15/12/2004 | 05:21:46 | 1.5 | 07:38:14 | 253 |
|  |  | 25/12/2004 | 06:37:27 | 0.8 | 07:59:15 | 105 |
|  |  | 01/01/2005 | 16:58:13 | 1.4 | 19:01:15 | 131 |
|  |  | 11/01/2005 | 21:34:30 | 0.8 | 23:05:44 | 151 |
|  |  | 22/01/2005 | 16:06:17 | 0.9 | 17:29:02 | 124 |
|  |  | 28/01/2005 | 02:29:41 | 0.7 | 03:28:12 | 97 |
|  |  | 08/02/2005 | 20:44:24 | 0.6 | 20:59:54 | 121 |
|  |  | 14/02/2005 | 04:24:32 | 0.9 | 05:27:24 | 84 |
|  |  | 26/02/2005 | 04:32:40 | 0.4 | 05:14:31 | 74 |
|  |  | 28/02/2005 | 21:16:14 | 1.4 | 23:08:50 | 170 |
|  |  | 13/03/2005 | 12:18:53 | 0.5 | 13:10:57 | 66 |
|  |  | 24/03/2005 | 00:37:47 | 0.7 | 01:00:29 | 129 |
|  |  | 28/03/2005 | 11:29:55 | 0.3 | 10:29:17 | 32 |
|  | SW | 24/12/2002 | 12:55:30 | 0.7 | 13:54:56 | 40 |
|  |  | 01/01/2003 | 23:17:44 | 0.6 | 00:14:55 | 58 |
|  |  | 04/01/2003 | 23:16:35 | 0.7 | 23:50:41 | 95 |
|  |  | 09/01/2003 | 15:59:42 | 2.1 | 20:07:45 | 280 |
|  |  | 01/02/2003 | 05:51:35 | 1.4 | 07:53:03 | 129 |
|  |  | 08/02/2003 | 20:11:15 | 1.3 | 21:52:09 | 218 |
|  |  | 16/02/2003 | 09:36:04 | 2.0 | 12:52:25 | 242 |
|  |  | 05/03/2003 | 03:14:01 | 1.8 | 05:58:13 | 228 |
|  |  | 15/03/2003 | 01:54:57 | 0.7 | 02:55:15 | 84 |
|  |  | 20/03/2003 | 06:43:26 | 0.4 | 07:00:45 | 50 |
|  |  | 29/03/2003 | 21:24:21 | 1.2 | 23:37:11 | 85 |

*Supplementary file 1b. Position, recording equipment and approximate (Approx.) depth for each location sampled.*

| Regions | Location | Latitude and longitude (°) | Recording equipment (AR or OBS) | Aprox. depth (m) |
| --- | --- | --- | --- | --- |
| SE Greenland | SE Greenland | 60° N  35° W | AR(Fox et al., 2001) | 800 |
| SE Iceland | SE Iceland | 64.185° N  14.686° W | AR (Calupca et al., 2000) | 60 |
| Celtic Sea | North Porcupine | 52.6221° N  15.3045° W | AR  (AMARs, JASCO  Applied Sciences, Halifax, Canada) | 1700 |
|  | South Porcupine | 49.5478°N  13.3723°W |  |  |
| Oceanic Northeast Atlantic (ONA) | North East (NE) | 49.8554°N  25.4541°W | AR(Fox et al., 2001) | 4200 |
|  | North West (NW) | 47.5941°N  -32.4500°W |  | 4100 |
|  | Central East (CE) | 40.3365°N  25.0350°W |  | 3300 |
|  | Central West (CW) | 42.7188°N  34.7226°W |  | 3970 |
|  | Azores | 38.5396°N  29.0434°W |  | 900 |
|  |  |  | AR (EARs)(Lammers et al., 2008) | 200-400 |
|  | South East (SE) | 32° N  35° W | AR(Fox et al., 2001) | 926 |
|  | South West (SW) | 26°N  50°W |  |  |
| SW Portugal | SW Portugal | 35.7798°N  10.3584°W | OBS(Silva, 2017) | 1993-5100 |
|  |  | 36.5753° N  11.5969° W | AR (EARs)(Lammers et al., 2008) | 255 |
| Canary Islands | Canary Islands | 28.8997°N  13.2003°W | OBS(Ugalde et al., 2019) | 1350 |
| Barents Sea | Svalbard | 79.0536°N  11.5471°E | AR (SM2, Wildlife Acoustics, US) | 75 |
|  | Vesterålen | 68.9°N  14.3838°E | AR (SB35 ETH, Ocean Sonics) | 258 |

Supplementary file 1c. Description of fin whale song measurements made with Raven Pro 1.4 software (Charif et al., 2010).

| **Raven measurement name** | **Description** | **Units** |
| --- | --- | --- |
| **Begin time** | The time at which the selection begins. | Seconds |
| **End time** | The time at which the selection ends. | Seconds |
| **Time 5%** | The point in time that divides the selection into two-time intervals containing 5% and 95% of the energy in the selection. | Seconds |
| **Inband Power** | Integral of the average power spectral density (PSD) over the band of interest. Since Raven uses Digital Fourier Transform, it averages the PSD with respect to time, sums up the time-averaged PSD in all Fourier bins and multiplies by the bin size in Hertz. | Decibels |
| **Peak frequency** | The frequency at which Peak Power occurs within the  selection. If Peak Power occurs at more than one time and/or frequency, the lowest frequency at Max Time at which Peak Power occurs. | Hertz |

***Supplementary file 1d. The effect of recording equipment on fin whale song parameters***

When comparing data from multiple sensors an obvious question is whether the results might be dependent on the specific sensors considered. To investigate the influence of the acoustic recorder type, OBS or ARs, on the song parameters, we analysed the same song fragment, consisting of 209 notes, recorded by the hydrophone channel of an OBS and an AR, specifically an Ecological Acoustic Recorder (EAR)(Lammers et al., 2008). The two instruments were deployed at ~6 km from each other in the Azores region in spring of 2019. Measurements of INIs and 20-Hz peak frequencies of songs recorded by each instrument were compared using a non-parametric paired samples Wilcoxon Test. Differences in HF note peak frequencies could not be tested because of limitations in the sampling rate of the OBS. Results showed that median INIs measured from OBS (16.52) and EARs (16.42) were not significantly different (p-value = 0.47) but median peak frequencies of the 20-Hz note (median OBS = 23.4; EARs = 21.1) were (p-value < 0.001). Thus, the use of different recorders did not affect INI measurements but influenced measurements of 20-Hz peak frequencies. The effect of the distance to the source in the analysed fin whale song parameters is included in this study, given that these two recorders were positioned at different distances to the singer. Peak frequencies of the 20-Hz note showed a great variability between equipment types (Fig. S3), which hindered the identification of soft trends (i.e., low changing rate). For this reason, only data from the EARs, the longest dataset (2008 – 2020), were used to study temporal variations of the peak frequencies of the 20-Hz note. All statistical analyses were performed using the software R (v. 4.0.2)(R Core team, 2020).

**REFERENCES**

Calupca TA, Fristrup KM, Clark CW. 2000. A compact digital recording system for autonomous bio- acoustic monitoring. *Journal of Acoustic Society of America* **108**:2582–2582.

Charif RA, Waack AM, Strickman LM. 2010. Raven Pro 1.4 User’s Manual. The Cornell Lab of Ornithology.

Fox CG, Matsumoto H, Lau T-K. 2001. Monitoring Pacific Ocean seismicity from an autonomous hydrophone array Christopher. *J Geophys Res* **106**:4183–4206. doi:10.1533/9781782422723.41

Lammers MO, Brainard RE, Au WWL, Mooney TA, Wong KB. 2008. An ecological acoustic recorder (EAR) for long-term monitoring of biological and anthropogenic sounds on coral reefs and other marine habitats. *J Acoust Soc Am* **123**:1720–1728. doi:10.1121/1.2836780

R Core team. 2020. R: A language and environment for statistical computing. R Foundation for Statistical Computing, Vienna, Austria.

Silva SDMMF da. 2017. Strain partitioning and the seismicity distribution within a transpressive plate boundary: SW Iberia-NW Nubia. University of Lisbon.

Ugalde A, Gaite B, Fernández MR, Villaseñor A, Ranero CR. 2019. Seismicity and Noise Recorded by Passive Seismic Monitoring of Drilling Operations Offshore the Eastern Canary Islands. *Seismological Research Letters* **90**:1565–1576. doi:10.1785/0220180353
